# Annual change in FEV_1_ in elderly 10-year survivors with established chronic obstructive pulmonary disease

**DOI:** 10.1038/s41598-019-38659-8

**Published:** 2019-02-14

**Authors:** Masaru Suzuki, Hironi Makita, Satoshi Konno, Kaoruko Shimizu, Yasuyuki Nasuhara, Katsura Nagai, Yasushi Akiyama, Satoshi Fuke, Hiroshi Saito, Takeshi Igarashi, Kimihiro Takeyabu, Masaharu Nishimura

**Affiliations:** 1 Department of Respiratory Medicine, Faculty of Medicine and Graduate School of Medicine, Hokkaido University, Sapporo, Japan; 2 Center for Respiratory Diseases, JCHO Hokkaido Hospital, Sapporo, Japan; 3 Department of Internal Medicine, KKR Sapporo Medical Center, Sapporo, Japan; 4 Department of Internal Medicine, Hokkaido Chuo Rosai Hospital, Iwamizawa, Japan; 5 Department of Respiratory Medicine, Otaru Kyokai Hospital, Otaru, Japan

## Abstract

Long-term decline in lung function is generally considered to be progressive in individuals with established chronic obstructive pulmonary disease (COPD), despite the presence of intersubject variation. We hypothesized that the annualized rate of decline in forced expiratory volume in 1 second (FEV_1_) would not be constant among different time periods in the natural history of established COPD. We compared the annual change rates in FEV_1_ during the first 5 years and the last 5 years, estimated separately using a linear mixed-effects model in 10-year survivors (n = 110). The subjects were classified into three FEV_1_ decline groups, based on the 25th and 75th percentile values in each time period. The rates of FEV_1_ changes, calculated from the first 5 years and the last 5 years, did not correlate with each other among 10-year survivors; the subjects of each FEV_1_ decline group during the first 5 years did not consistently remain in the same FEV_1_ decline group during the last 5 years. Smoking status and exacerbation frequency were not associated with decline in FEV_1_. In conclusion, the disease activity, which is often expressed as annualized change in FEV_1_, might be changeable either way over years in patients with established COPD.

## Introduction

Chronic obstructive pulmonary disease (COPD) is a leading cause of morbidity and mortality worldwide^1^. Therefore, better understanding of the long-term natural history of COPD is needed to facilitate better management and intervention strategies for the disease. Since the pioneering studies by Fletcher and Peto and coworkers^2,3^, forced expiratory volume in 1 second (FEV_1_) has been thought to deteriorate progressively in patients with COPD; notably, FEV_1_ determines disease severity based on airflow limitation^1^. In addition to disease severity, the concept of disease activity is important for characterizing COPD. Disease activity in COPD comprises several aspects, including frequent exacerbations or loss of quality of life; the rate of decline in FEV_1_ should be considered one of the most vital aspects of disease activity because airflow limitation is essential to the nature of COPD. Such progression of airflow limitation actually involves contribution of various pathological processes, such as inflammation in both small and large airways as well as emphysema progression. Indeed, annualized change in FEV_1_ has often been used as vital outcome measures in several landmark, large-scaled, randomized controlled trials^4,5^.

Several observational cohort studies have recently shown wide interindividual variability with respect to changes in FEV_1_ among patients with COPD; notably, some patients have exhibited sustained lung function over several years^6–8^. Furthermore, the Lung Health Study and several general population-based studies have shown that FEV_1_ does not decrease in linear fashion; in contrast, its trajectories are variable^9–13^. However, it is unclear whether the long-term annualized rate of change in FEV_1_ is relatively constant or variable in individuals with established COPD that is appropriately managed. Thus far, there have been no studies that involved intraindividual comparison of the annualized rate of FEV_1_ decline across different time periods. We hypothesized that the annualized rate of FEV_1_ decline (i.e., the disease activity characterized by the rate of lung function decline) does not remain constant across different time periods during the clinical course of COPD.

In this study of 10-year survivors, we compared annual change rates in FEV_1_ during the first 5 years and the last 5 years; we used the data from the Hokkaido COPD cohort study, which was prospectively planned and carefully conducted with a high follow-up rate^8,14–16^. Our goal with regard to focusing on 10-year survivors was that we anticipated characterization of long-term individual lung function decline in patients with established COPD that was appropriately managed. In addition, annualized changes in FEV_1_ were calculated using separate spirometric data for 5-year’ periods in order to increase the accuracy of estimates for the annualized rate. Because exacerbation events were infrequent and were not associated with annualized decline patterns in FEV_1_ in the Hokkaido COPD cohort study^15^, this cohort is suitable for examination of the natural history of lung function without modification by exacerbation events.

## Results

### Ten-year survivors in the Hokkaido COPD cohort study

**Figure 1:**
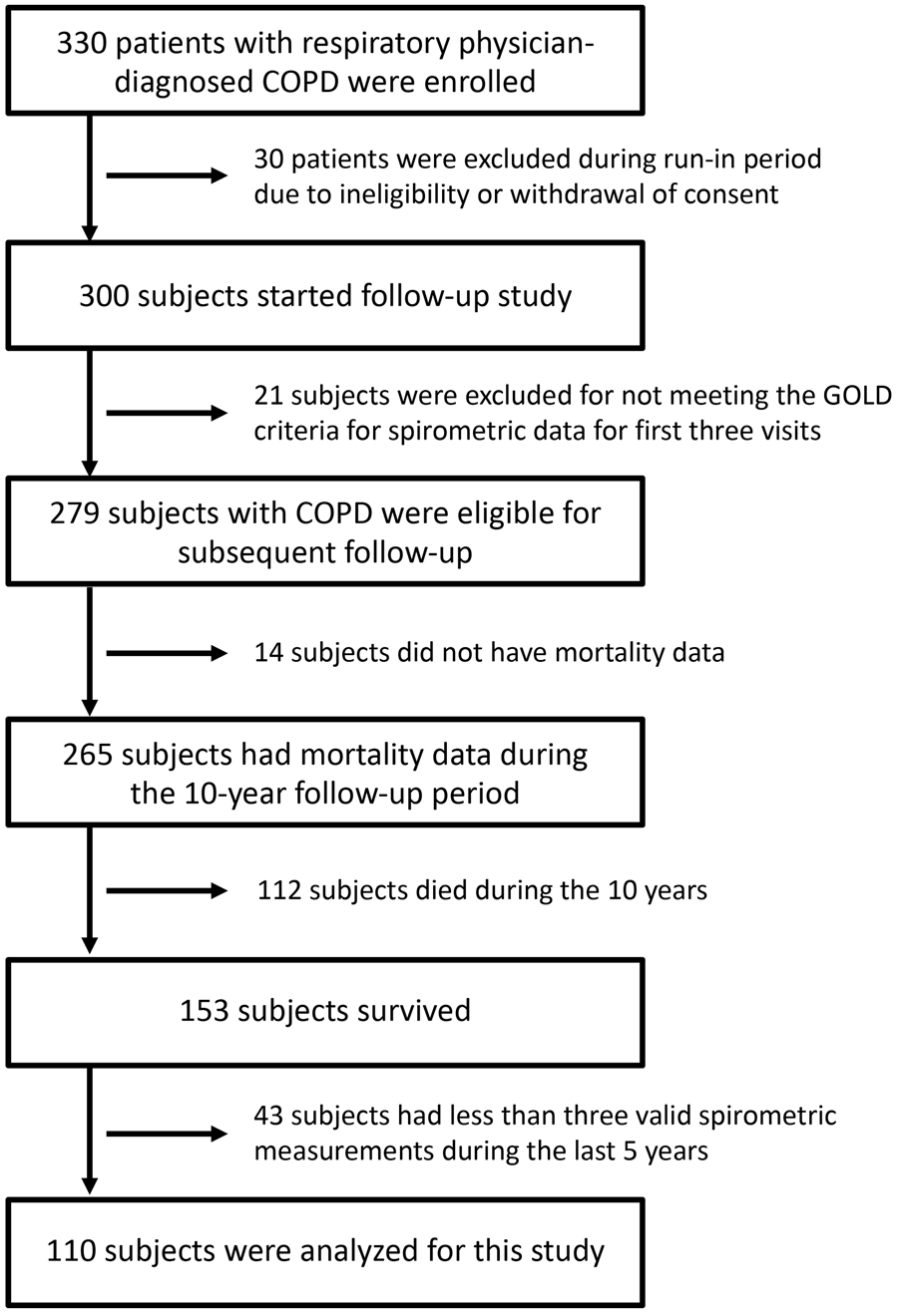
Flow chart of subject selection.

Figure 1 shows the flow chart of subject selection for this study. Of 279 subjects with spirometry-confirmed COPD in the first year, 265 (95%) had mortality data during the 10-year follow-up period; 153 (55%) survived and 112 (40%) died. Among the 153 10-year survivors, 110 subjects (72%) had at least three valid spirometric measurements during the last 5 years (10-year survivors with good follow-up), whereas 43 subjects (28%) had less than three valid spirometric measurements (10-year survivors lost to follow-up). In this study, 10-year survivors with good follow-up (110 subjects) were analyzed; their characteristics, in comparison with other groups, are shown in Table 1. Ten-year survivors were significantly younger, had a higher body mass index (BMI), better lung function, and a lower CT emphysema score than non-survivors, regardless of spirometric follow-up status (Table 1). Exacerbation frequency during the first 5 years did not statistically differ among the three groups (Table 1).

**Table 1:** Characteristics of 10-year survivors and non-survivors.

	10-year survivors with good follow-up	10-year survivors lost to follow-up	10-year non-survivors	P value
Number of subjects Baseline variables	110	43	112	
Age, years	66 ± 7*	68 ± 9*	74 ± 5	<0.001^a^
Female sex, N (%)	7 (6)	4 (9)	3 (3)	0.16^c^
BMI, kg/m^2^	23 ± 3*	23 ± 3*	21 ± 3	0.001^a^
Current smoker at entry, N (%)	30 (27)	15 (35)	28 (25)	0.46^c^
Smoking index at entry, pack-years	66 ± 32	69 ± 33	58 ± 25	0.07^a^
Lung function				
Post-BD FEV_1_, L	1.91 ± 0.65*	1.81 ± 0.72	1.54 ± 0.61	<0.001^a^
Post-BD FEV_1_, % predicted	68 ± 20*	69 ± 27*	59 ± 21	0.004^a^
Post-BD FVC, % predicted	103 ± 16	103 ± 25	98 ± 18	0.15^a^
Post-BD FEV_1_/FVC	0.54 ± 0.13*	0.53 ± 0.13	0.48 ± 0.12	0.001^a^
Reversibility of FEV_1_, %	12 ± 11	9 ± 10	12 ± 11	0.25^a^
Reversibility of FEV_1_, mL	167 ± 110^†^	118 ± 96	135 ± 105	0.01^a^
DLco, % predicted	81 ± 23	80 ± 26	73 ± 26	0.07^a^
Kco, % predicted	70 ± 23*	63 ± 22	57 ± 24	<0.001^a^
Patient-reported outcomes				
Chronic bronchitis, N (%)	12 (11)	5 (12)	12 (11)	1.00^c^
mMRC dyspnea score ≥ 2, N (%)	51 (46)	24 (56)	68 (61)	0.10^c^
SGRQ total score	30 ± 16	31 ± 18	34 ± 18	0.13^a^
Laboratory values				
Blood neutrophil count, cells/mm^3^	3394 (2629–4038)	3479 (2967–4135)	3505 (2736–4054)	0.65^b^
Blood eosinophil count, cells/mm^3^	176 (101–308)	159 (116–244)	160 (87–250)	0.34^b^
Serum total IgE, IU/mL	82 (23–223)	65 (20–119)	63 (19–180)	0.28^b^
CT emphysema score	1.0 (0.5–1.7)*	1.3 (0.5–2.2)	1.5 (0.8–2.3)	<0.001^b^
Comorbidities				
Any cardiovascular disease	22 (20)	8 (19)	29 (26)	0.50^c^
Ischemic heart disease	8 (7)	2 (5)	7 (6)	0.89^c^
Diabetes	5 (5)	3 (7)	5 (4)	0.80^c^
Charlson risk index	0(0–1)	0(0–0)	0(0–1)	0.29^b^
Longitudinal variables (first 5 years) Exacerbation frequency, events/y	0 (0–0.20)	0(0–0)	0 (0–0.40)	0.09^b^

14 subjects whose 10-year survival data were absent were not included in this table.

Data are shown as means ± SD, median (interquartile range), or number (%).

Post-BD = post-bronchodilator; DLco = carbon monoxide diffusion capacity; Kco = carbon monoxide transfer coefficient.

Reversibility of FEV_1_, % refers to % change in FEV_1_ by inhalation of bronchodilator.

^a^One-way analysis of variance, ^b^Kruskal-Wallis test, ^c^Fisher’s exact test.

*p < 0.05 vs. 10-year non-survivors, ^†^p < 0.05 vs. 10-year survivors lost to follow-up (Tukey’s HSD test or Mann-Whitney U test).

### Ten-year lung function change in subjects with COPD

**Figure 2:**
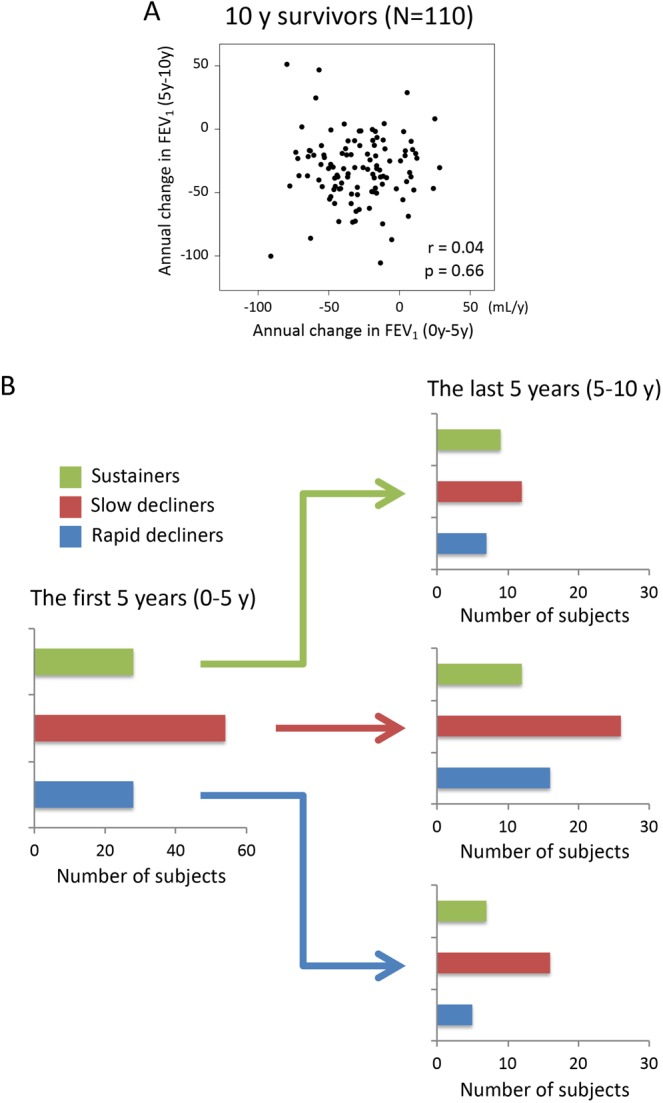
Correlations between annual changes in FEV_1_ calculated from the first 5 years and the last 5 years among 10-year survivors with good follow-up (n = 110). (**A**) Correlation between annual changes in FEV_1_ of the first 5 years (0–5 y) vs. the entire 10 years (0–10 y). (**B**) Bar plots of the number of subjects in each FEV_1_ decline group for the first 5 years and the last 5 years. The bars on the left show the number of subjects in each FEV_1_ group (rapid decliners, slow decliners, and sustainers) for the first 5 years. The bars on the right show the respective numbers of subjects in each FEV_1_ group for the last 5 years.

**Figure 3:**
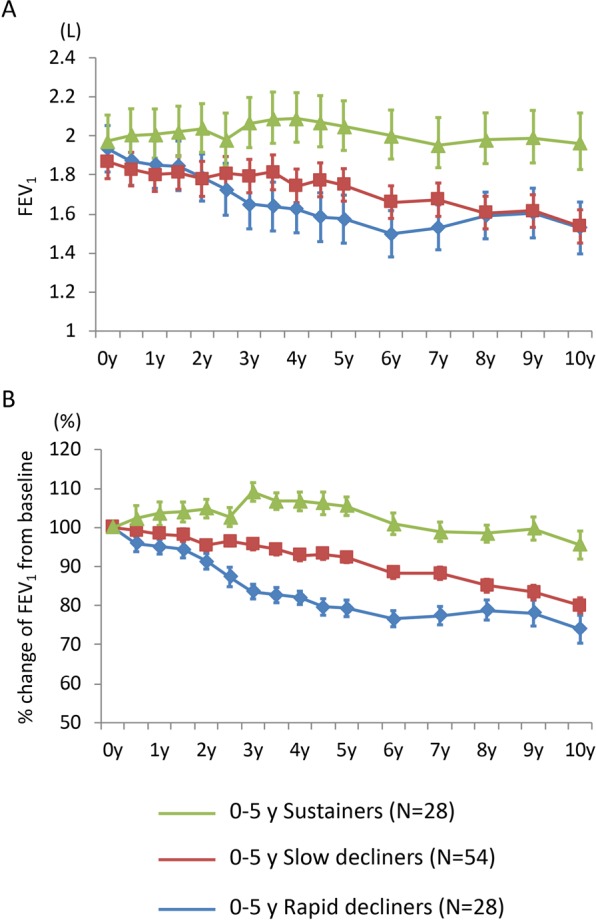
10-year annual change in FEV_1_ among FEV_1_ decline groups for the first 5 years. (**A**) Mean post-bronchodilator FEV_1_ (with SEM) expressed as absolute values. (**B**) Mean post-bronchodilator FEV_1_ (with SEM) expressed as percent changes from baseline.

Among 10-year survivors with good follow-up, the median number of spirometric assessments per subject was 16 (IQR: 15-16) during the entire 10-year period, 11 (IQR: 11-11) during the first 5 years, and 5 (IQR: 5-5) in the last 5 years, respectively. Their calculated annual change in post-bronchodilator FEV_1_ was −31 ± 21 mL/year during the entire 10-year period (“rapid decliners”: −57 ± 15 mL/year, “slow decliners”: −31 ± 9 mL/year, “sustainers”: −6 ± 11 mL/year), −28 ± 25 mL/year during the first 5 years (rapid decliners: −61 ± 11 mL/year, slow decliners: −29 ± 10 mL/year, sustainers: 4 ± 11 mL/year), and −30 ± 28 mL/year during the last 5 years (rapid decliners: −62 ± 16 mL/year, slow decliners: −31 ± 9 mL/year, sustainers: 3 ± 22 mL/year) (Fig. S1A–C). Of note, there was no significant correlation between annual changes in post-bronchodilator FEV_1_ during the first 5 years and the last 5 years (r = 0.04, p = 0.66) (Fig. 2A). In addition, the subjects of each FEV_1_ decline group during the first 5 years were recategorized to any of the three decline groups during the last 5 years (Fig. 2B). Chronologic changes in the mean FEV_1_, and those expressed as percent changes from baseline in each FEV_1_ decline group during the 10-year period, are shown in Fig. 3 (FEV_1_ decline group during the first 5 years) and Fig. S2 (FEV_1_ decline group during the last 5 years). In particular, rapid decliners during the first 5 years showed much slower FEV_1_ decline after 5 years; their FEV_1_ reached levels similar to those of slow decliners after 8 years of follow-up (Fig. 3). When GOLD airflow limitation grade at baseline was considered, rapid decliners during the first 5 years who were GOLD 2 or more at baseline showed slower FEV_1_ decline after 5 years; those who were GOLD 1 continued to exhibit FEV_1_ decline after 5 years (Fig. S3). These results clearly indicate that the rate of lung function decline can change in either direction, regardless of GOLD stage, in the natural history of COPD.

Next, we investigated factors associated with the rate of FEV_1_ decline. Among 10-year survivors with good follow-up, rapid FEV_1_ decline during the entire 10-year period was associated with lower BMI, lower diffusion capacity, and a lower blood eosinophil count at baseline (Table S1). Notably, the degree of FEV_1_ decline during the last 5 years was not associated with any variables at the 5-year follow-up (Table S2). Smoking status and exacerbation frequency during the first 5 years were similar among the FEV_1_ decline groups in each condition (Tables S1 and S2).

## Discussion

Progressive airflow limitation (reduction of FEV_1_) is an essential feature of COPD, and the percent predicted value for FEV_1_ determines disease severity as a result of small airway disease and emphysema^1^. Assessment of FEV_1_ as an index of disease severity is important for predicting the future clinical course of COPD patients, including morbidity and mortality at the population level^1^. One of the primary treatment goals for COPD patients is effective prevention of disease progression. To achieve this goal, assessment of disease activity is very important. Both disease severity and activity are associated with pathological processes but they are distinct concepts, and disease severity cannot be a surrogate for disease activity^14^. The concept of disease activity is essential for the management of chronic inflammatory diseases, such as rheumatoid arthritis and inflammatory bowel diseases^15,16^; however, individual chronological changes in disease activity based on the rate of lung function decline in established COPD patients have not been fully investigated. Because the rate of FEV_1_ decline is regarded as a vital measure of COPD disease activity, annualized changes in FEV_1_ were compared during different time periods in the same individuals with COPD. In the present study, annualized changes in FEV_1_ were calculated using separate spirometric data for 5-year periods, in order to increase the accuracy of estimates for the annualized rate. Notably, measurement of the true rates of FEV_1_ decline requires numerous time points over many years of follow-up, due to the relatively high variability in FEV_1_. Furthermore, the inter-subject variation in calculated rates of annual decline in FEV_1_ is smaller with longer follow-up periods^17^. In the present study, spirometric measurements were performed every 6 months during the first 5 years and every year afterward, based on scheduled visits; indeed, the median number of spirometric assessments was 16 during the 10-year period. Furthermore, we confirmed that the subjects were clinically stable at each visit.

In agreement with our hypothesis, the rates of FEV_1_ changes calculated from the first 5 years and from the last 5 years were not correlated among 10-year survivors; the subjects of each FEV_1_ decline group during the first 5 years did not consistently remain in the same FEV_1_ decline group during the last 5 years (Fig. 2A,B). These findings indicate that COPD disease activity, defined by the rate of decline in lung function, is likely to change in each individual over a long-term period. Previous clinical trials showed that patients with milder airflow limitation had more rapid average decline in FEV_1_, compared with those with more severe airflow limitation^18^; this suggests that the loss of lung function is more accelerated in the initial phases of COPD. The present study extends this finding by showing that such variable rates of lung function decline were present in individuals at each GOLD grade (i.e., disease severity) (Fig. S3). Disease severity in each COPD patient must be a result of different lung function trajectories, including reduced lung function in early adulthood; accelerated decline in FEV_1_ is not an obligatory feature of COPD^12,13^. Therefore, we must consider disease severity, as well as disease activity (characterized by the rate of lung function decline), in the management of COPD.

In the present study, lower BMI, lower diffusion capacity, and lower blood eosinophil count at baseline were associated with a rapid decline in FEV_1_ during the entire 10-year period (Table S1), which is consistent with our previous report based on the data from the first 5 years^8^. Of note, in the 10-year survivors, none of the variables at the 5-year follow-up was associated with the rate of FEV_1_ decline during the last 5 years (Table S2). This observation suggests that the factors associated with the rate of lung function decline may change depending on the time point during the clinical course of COPD in 10-year survivors. For example, patients with severe emphysema who experienced rapid FEV_1_ reduction during the first 5 years would not exhibit further reduction during the last 5 years because of their low absolute FEV_1_ values. In this case, patients with severe emphysema would resemble rapid decliners during the first 5 years, and would then resemble sustainers during the last 5 years. Another explanation might be that unknown risk factors affect future lung function decline in 10-year survivors.

Importantly, exacerbation frequency was not associated with future lung function decline in the present study. The reduced exacerbation frequency in the Hokkaido COPD cohort study, compared with some large-scale clinical trials in the Western countries, may be responsible for the absence of a significant association between exacerbation frequency and lung function decline^19^. Alternatively, exacerbation may represent a different aspect of COPD disease activity, independent of natural lung function decline. We previously reported that the neutrophil elastase-alpha1-protenase inhibitor (NE-alpha1PI) complex in bronchoalveolar lavage fluid was markedly elevated in asymptomatic smokers who had subclinical emphysema on CT scans^20^; furthermore, higher levels of the NE-alpha1PI complex were associated with accelerated decline in FEV_1_^21^. This suggests that lung function decline could be affected by local proteolytic conditions in the lung without apparent exacerbation events. Of course, exacerbation history and symptoms are important measures for COPD disease activity, as emphasized in current GOLD guidelines^1^; however, the present study clearly indicates that the rate of lung function decline should be assessed as an important metric of COPD disease activity.

In the present study, smoking status at baseline and during the first 5 years was not associated with future changes in lung function; in contrast, the Lung Health Study showed that smoking cessation reduced FEV_1_ decline in smokers with mild airway limitation^9,10^. This discrepancy may be due to differences in study population characteristics. In the present study, current smokers at baseline comprised only 27% of the study population; therefore, the effect of smoking cessation on FEV_1_ decline would be much weaker than in the Lung Health Study.

Although the sample size in this study was small relative to that of several previous large-scale observational cohort studies, the strongest point was that it was very carefully designed and performed; thus, it was possible to collect very accurate annual spirometric data for a 10-year period with a very low dropout rate. However, this study has some other limitations. First, all subjects were Japanese; therefore, future studies with subjects of other ethnic backgrounds are warranted. Second, exacerbation data, which might affect lung function decline, was not collected during the last 5 years. Third, subjects were at different stages during their natural histories of COPD at the time of enrollment (staggered entry). This limitation is inevitable because of the nature of the observational cohort study; however, we followed subjects for a sufficient period of time (10 years) and found that the chronological changes in the rate of lung function decline were present among all spirometric GOLD grades. Lastly, there may be a survivorship bias in the analysis of this study because only 10-year survivors with good follow-up were analyzed. We acknowledge this limitation; however, we firmly believe that focusing on 10-year survivors alone is valuable and provides clues regarding the long-term natural history of established COPD.

In summary, the disease activity, which is often expressed as annualized change in FEV_1_, may change in either direction in the natural history of patients with established COPD, regardless of spirometric GOLD stage. These findings challenge the current concept that COPD is a disease with a natural history characterized by relatively constant progressive decline in FEV_1_. In addition to disease severity, disease activity (characterized by the rate of lung function decline) should also be considered in the management of COPD, as well as within any clinical research regarding COPD.

## Methods

### Participants

The cohort details have been described elsewhere^8,19,22,23^. Briefly, a total of 330 Japanese patients with COPD were recruited at Hokkaido University Hospital, Sapporo, Japan, and its nine affiliated hospitals from May 2003 to May 2005. Subjects with clinically-diagnosed asthma were meticulously excluded^23^. During the first follow-up year (visits 1–3), the diagnosis was reconfirmed based on the spirometric criteria of the Global Initiative for Chronic Obstructive Lung Disease (GOLD) guidelines (a ratio of post-bronchodilator FEV_1_ to FVC < 0.70)^1^. As a result, a total of 279 subjects with COPD (GOLD 1, 26%; GOLD 2, 45%; GOLD 3, 24%; and GOLD 4, 5%) were eligible for subsequent follow-up. The Ethics Committee of Hokkaido University School of Medicine approved the study protocol (med02-001) and written informed consent was obtained from all participants. This study was performed in accordance with the Declaration of Helsinki.

### Study protocol

Until the fifth year, information regarding COPD exacerbations was collected every month. Spirometry before and after short-acting bronchodilator inhalation was performed every 6 months after confirming withdrawal of any long-acting bronchodilators. Diffusion capacity testing, chest computed tomography (CT), and a health-related quality of life assessment using St. George’s Respiratory Questionnaire (SGRQ) were performed each year. The severity of emphysema on chest CT was assessed visually^8,22,24^. Exacerbation was defined as worsening or new-onset of respiratory symptoms that required changes in prescription treatment^19^.

After the fifth year, spirometry after bronchodilator inhalation and diffusion capacity testing was performed every year for those who agreed with the extension of the scheduled follow-up program until the tenth year. Spirometry data after the diagnosis of lung cancer or any major comorbidities, which might interfere with the natural history of COPD, were not used in this study. The majority of the subjects continued to visit outpatient clinics for appropriate medical care, even if they dropped out from the scheduled follow-up program of this study. Therefore, a telephone interview and/or medical chart review to monitor annual mortality data over 10 years was required in only a minority of the study population.

### Statistical analysis

Annual changes in post-bronchodilator FEV_1_ during the first 5 years, the last 5 years, and the entire 10-year period were estimated separately using a linear mixed-effects model. In each time period, the subjects were classified into three FEV_1_ decline groups based on the magnitude of the annual change in FEV_1_: rapid decliners, those in the less than 25th percentile group; slow decliners, those in the 25th to 75th percentile group; and sustainers, those in the greater than 75th percentile group^8^. Correlations between annual changes in FEV_1_ during the two time periods were analyzed using Pearson’s correlation coefficient. Differences among the groups were analyzed using one-way analysis of variance, the Tukey’s HSD test, the Kruskal-Wallis test, the Mann-Whitney U test, or Fisher’s exact test, as appropriate. Statistical significance was defined as p < 0.05. All analyses were performed using R version 3.1.2 (The R Foundation, http://www.r-project.org/).

## Supplementary information

Online data supplement
