# Supplementary material for: Annual change in FEV1 in elderly 10-year survivors with established chronic obstructive pulmonary disease
Source: Sci Rep. 2019 Feb 14;9:2073. doi: 10.1038/s41598-019-38659-8 (PMC6375910; doi:10.1038/s41598-019-38659-8)
Supplement: Supplementary file 1 — Online data supplement [file 41598_2019_38659_MOESM1_ESM.pdf]

Annual change in FEV_1 in elderly 10-year survivors with established chronic obstructive pulmonary disease

Masaru Suzuki, Hironi Makita, Satoshi Konno, Kaoruko Shimizu, Yasuyuki Nasuhara, Katsura Nagai, Yasushi Akiyama, Satoshi Fuke, Hiroshi Saito, Takeshi Igarashi, Kimihiro Takeyabu, Masaharu Nishimura

Online data supplement

Table S1. Characteristics of the FEV_1 decline group during the entire-10 year period among 10-year survivors with good follow-up

	0-10y Rapid decliners	0-10y Slow decliners	0-10y Sustainers	P value
Number of subjects	28	54	28	
0-10 y Annual post-BD FEV_1 change, mL/y	-57 ± 15	-31 ± 9	-6 ± 11	
Baseline variables				
Age, years	67 ± 6	65 ± 7	67 ± 8	0.61[1]
Female sex, N (%)	0 (0)	6 (11)	1 (4)	0.15[3]
BMI, kg/m^2	21 ± 3	23 ± 3	25 ± 3	<0.001[1]
Current smoker, N (%)	6 (21)	16 (30)	8 (29)	0.79[3]
Lung function				
Post-BD FEV_1, L	1.95 ± 0.67	1.91 ± 0.60	1.87 ± 0.75	0.89[1]
Post-BD FEV_1, % predicted	68 ± 21	69 ± 20	66 ± 21	0.81[1]
DLco, % predicted	72 ± 24	85 ± 22	82 ± 19	0.04[1]
Kco, % predicted	55 ± 21	74 ± 22	77 ± 22	<0.001[1]
SGRQ total score	30 ± 19	29 ± 16	31 ± 14	0.85[1]
Laboratory values				
Blood neutrophil count, cells/mm^3	3542 (2788-4279)	3398 (2460-3896)	3098 (2859-3780)	0.67[2]
Blood eosinophil count, cells/mm^3	141 (88-192)	181 (118-342)	247 (118-342)	0.05[2]
Longitudinal variables during the first 5 years				
Smoking status, current/intermittent/ex	2/6/20	10/11/33	5/3/20	0.52[3]
Exacerbation frequency, events/y	0 (0-0.25)	0 (0-0.20)	0.10 (0-0.40)	0.58[2]
Pharmacotherapy				
Anticholinergics	12 (43)	25 (46)	8 (29)	0.33[3]
β_2-receptor agonists	7 (25)	18 (33)	10 (36)	0.74[3]
Inhaled corticosteroids	0 (0)	10 (19)	3 (11)	0.03[3]

Data are shown as means ± SD, median (interquartile range), or number (%). Post-BD = post-bronchodilator; DLco = carbon monoxide diffusion capacity; Kco = carbon monoxide transfer coefficient. [1] One-way ANOVA, [2] Kruskal-Wallis test, [3] Fisher's exact test.

Table S2. Characteristics of the FEV_1 decline group during the last 5 years among 10-year survivors with good follow-up

	5-10y Rapid decliners	5-10y Slow decliners	5-10y Sustainers	P value
Number of subjects	28	54	28	
5-10 y Annual post-BD FEV_1 change, mL/y	-62 ± 16	-31 ± 9	3 ± 22	
Variables at 5 years				
Age, years	71 ± 8	71 ± 7	71 ± 6	0.99[1]
Female sex, N (%)	1 (4)	5 (9)	1 (4)	0.68[3]
BMI, kg/m^2	22 ± 3	23 ± 3	24 ± 3	0.25[1]
Current smoker, N (%)	7 (25)	6 (11)	5 (18)	0.25[3]
Lung function				
Post-BD FEV_1, L	1.88 ± 0.57	1.70 ± 0.67	1.86 ± 0.70	0.41[1]
Post-BD FEV_1, % predicted	69 ± 13	65 ± 24	69 ± 23	0.64[1]
DLco, % predicted	84 ± 21	77 ± 30	74 ± 22	0.35[1]
Kco, % predicted	68 ± 19	65 ± 25	65 ± 20	0.85[1]
SGRQ total score	25 ± 17	27 ± 18	24 ± 17	0.75[1]
Laboratory values				
Blood neutrophil count, cells/mm^3	3788 (2964-4625)	3455 (2815-4163)	3328 (2775-3968)	0.43[2]
Blood eosinophil count, cells/mm^3	179 (122-218)	177 (101-250)	192 (113-268)	0.61[2]
Longitudinal variables during the first 5 years				
Smoking status, current/intermittent/ex	4/6/18	7/11/36	6/3/19	0.72[3]
Exacerbation frequency, events/y	0 (0-0.20)	0 (0-0.40)	0 (0-0.20)	0.72[2]
Pharmacotherapy				
Anticholinergics	9 (32)	26 (48)	10 (36)	0.37[3]
β_2-receptor agonists	10 (36)	16 (30)	9 (32)	0.83[3]
Inhaled corticosteroids	2 (7)	8 (15)	3 (11)	0.64[3]

Data are shown as means ± SD, median (interquartile range), or number (%). Post-BD = post-bronchodilator; DLco = carbon monoxide diffusion capacity; Kco = carbon monoxide transfer coefficient. [1] One-way analysis of variance, [2] Kruskal-Wallis test, [3] Fisher's exact test.

Figure legends

Figure S1. Histograms of annual changes in FEV_1 calculated from different time periods among 10-year survivors with good follow-up (n=110)

(A) The entire 10-year period (0-10 y) among 10-year survivors with good follow-up. (B) The first 5 years (0-5 y) among 10-year survivors with good follow-up. (C) The last 5 years (5-10 y) among 10-year survivors with good follow-up. In each figure, 1st quartile and 3rd quartile values are indicated by red lines.

Figure S2. 10-year annual change in FEV_1 among FEV_1 decline groups for the last 5 years

(A) Mean post-bronchodilator FEV_1 (with SEM) expressed as absolute values. (B) Mean post-bronchodilator FEV_1 (with SEM) expressed as percent changes from baseline.

Figure S3. 10-year annual change in FEV_1 among FEV_1 decline groups for the first 5 years with GOLD airflow limitation grades at baseline

Mean post-bronchodilator FEV_1 (with SEM) expressed as absolute values.

Figure S1

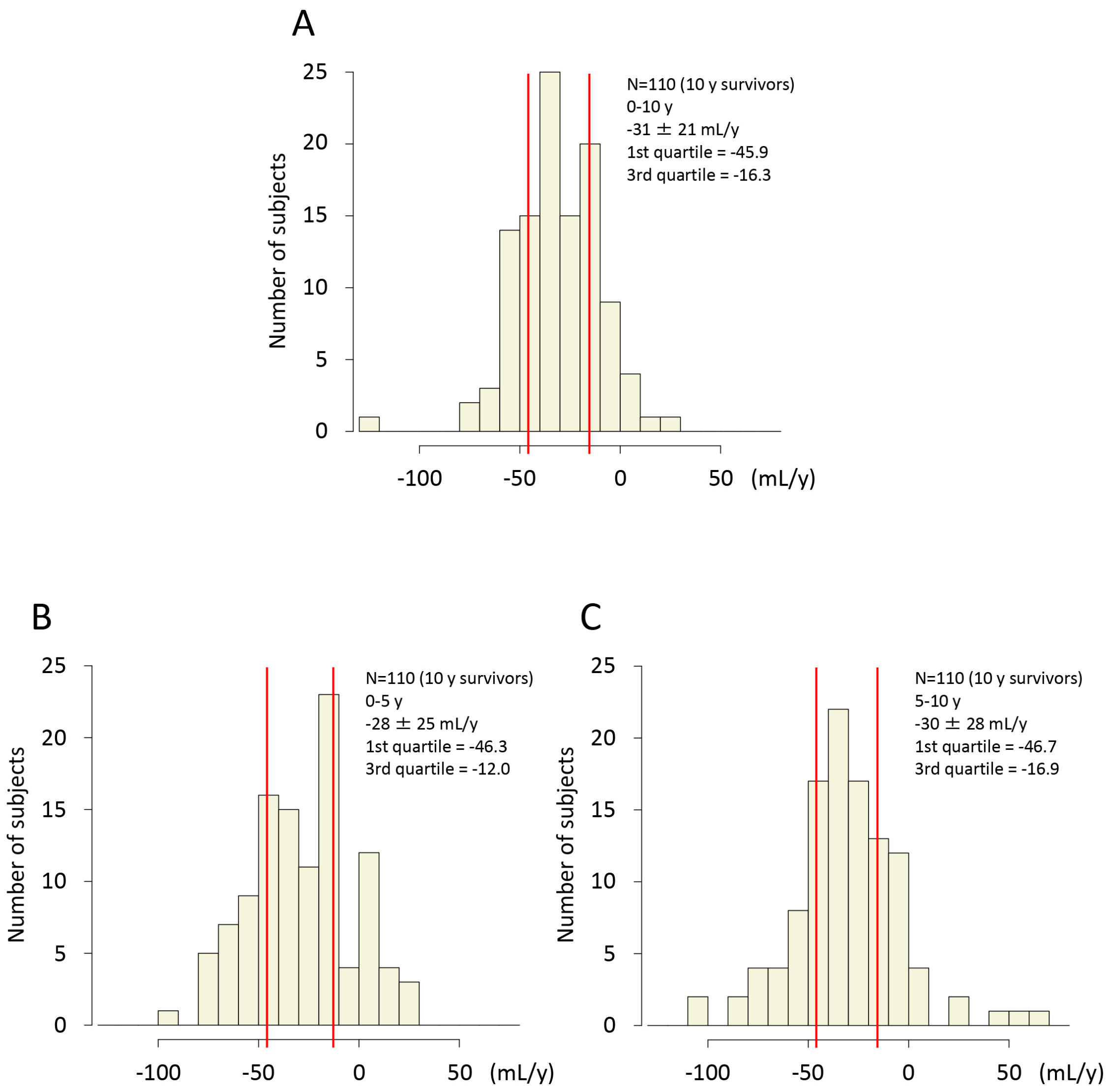

A
N=110 (10 y survivors)
0-10 y
-31 ± 21 mL/y
1st quartile = -45.9
3rd quartile = -16.3
Number of subjects
(mL/y)
B
N=110 (10 y survivors)
0-5 y
-28 ± 25 mL/y
1st quartile = -46.3
3rd quartile = -12.0
Number of subjects
(mL/y)
C
N=110 (10 y survivors)
5-10 y
-30 ± 28 mL/y
1st quartile = -46.7
3rd quartile = -16.9
Number of subjects
(mL/y)

Figure S2

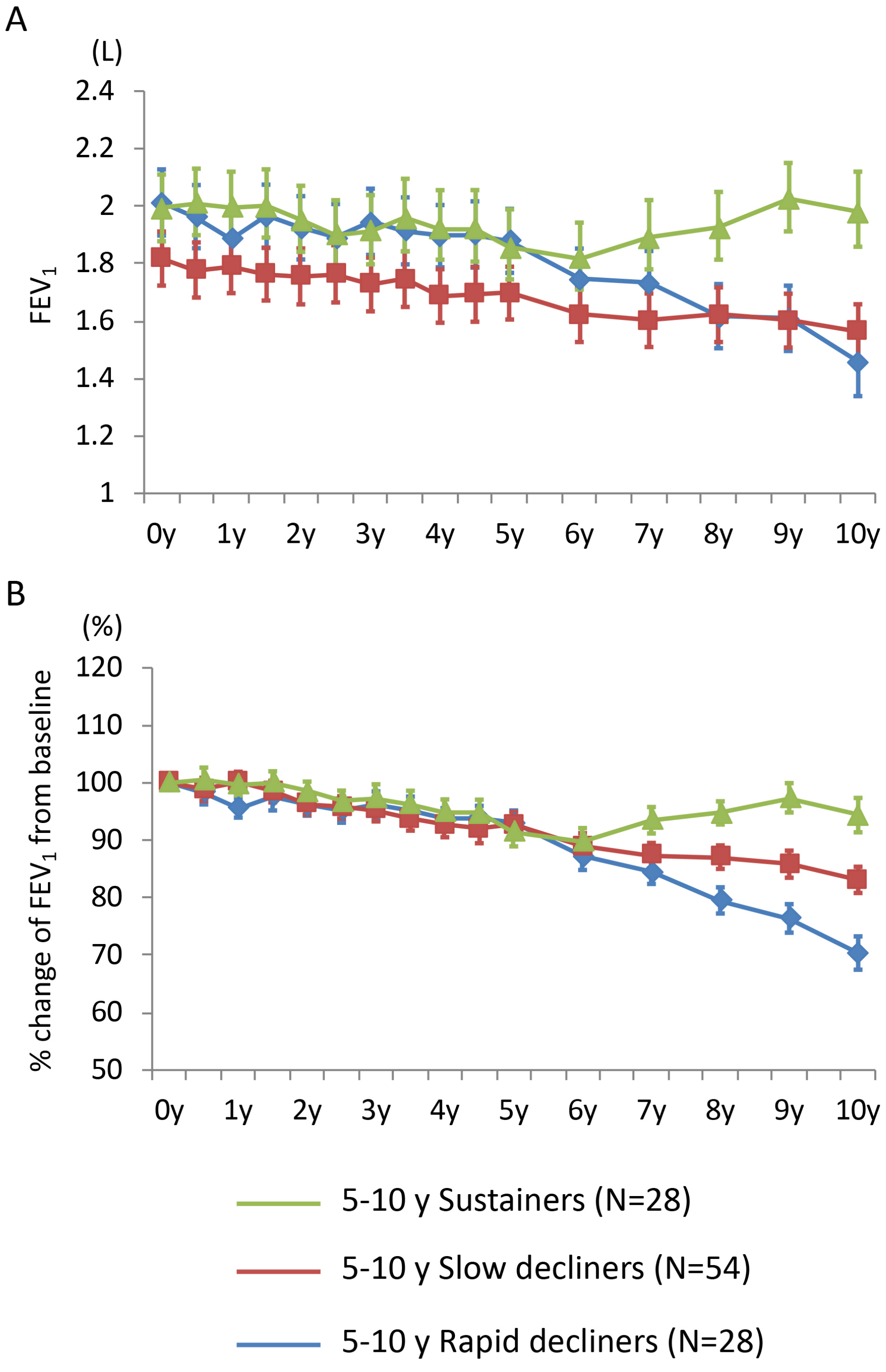
A
(L)
2.4
2.2
2
1.8
1.6
1.4
1.2
1
FEV1
0y
1y
2y
3y
4y
5y
6y
7y
8y
9y
10y
B
(%)
120
110
100
90
80
70
60
50
% change of FEV1 from baseline
0y
1y
2y
3y
4y
5y
6y
7y
8y
9y
10y
5-10 y Sustainers (N=28)
5-10 y Slow decliners (N=54)
5-10 y Rapid decliners (N=28)

Figure S3

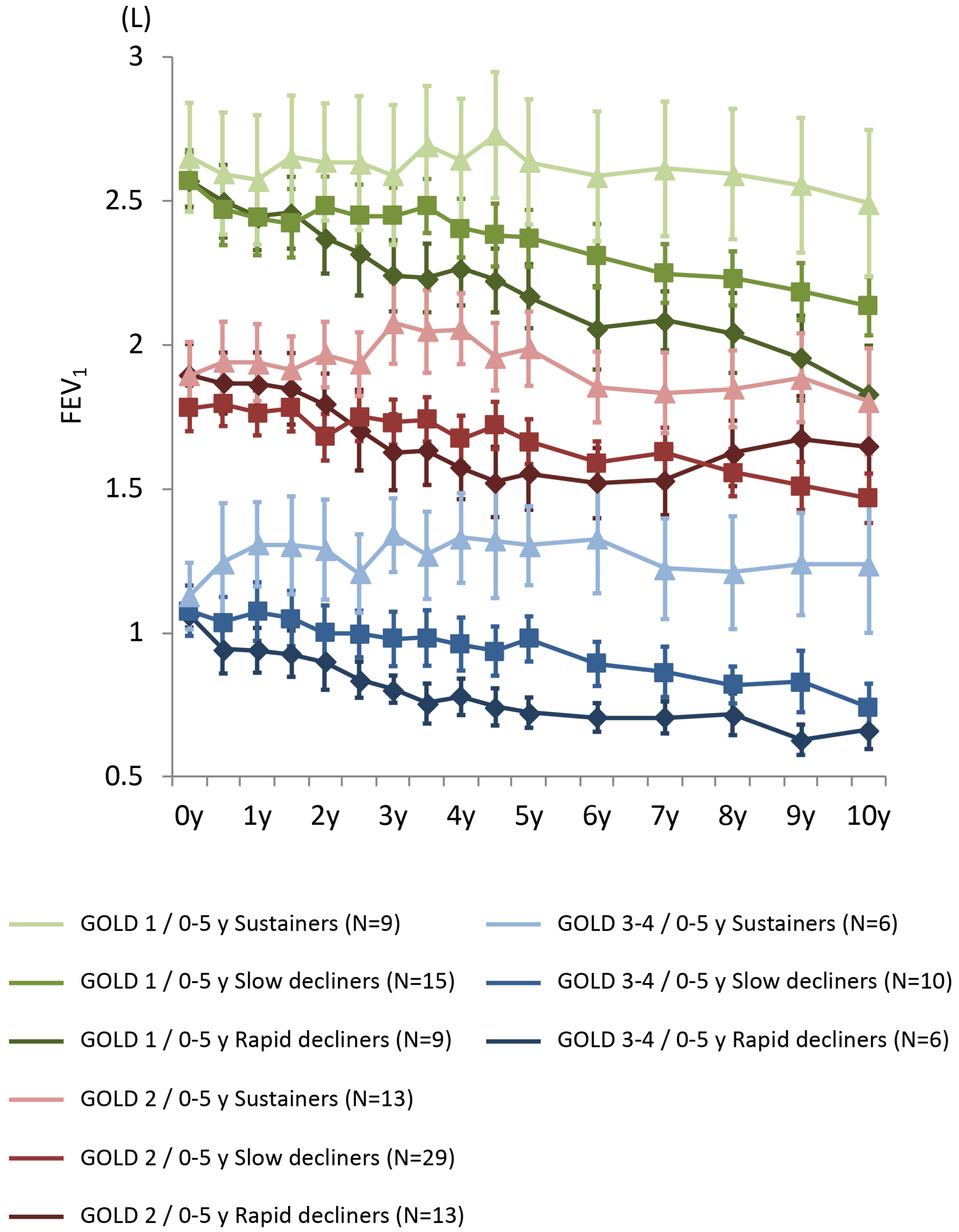

(L)
3
2.5
2
1.5
1
0.5
FEV1
0y
1y
2y
3y
4y
5y
6y
7y
8y
9y
10y
GOLD 1 / 0-5 y Sustainers (N=9)
GOLD 1 / 0-5 y Slow decliners (N=15)
GOLD 1 / 0-5 y Rapid decliners (N=9)
GOLD 2 / 0-5 y Sustainers (N=13)
GOLD 2 / 0-5 y Slow decliners (N=29)
GOLD 2 / 0-5 y Rapid decliners (N=13)
GOLD 3-4 / 0-5 y Sustainers (N=6)
GOLD 3-4 / 0-5 y Slow decliners (N=10)
GOLD 3-4 / 0-5 y Rapid decliners (N=6)
